# Strength and Push Gait Asymmetry in Skeleton Athletes

**DOI:** 10.5114/jhk/193480

**Published:** 2024-12-19

**Authors:** Min Gong, Yan Liu, Zhi Cao, Binghong Gao

**Affiliations:** 1 School of Sport Training, Guangzhou Sport University, Guangzhou, China.; 2 School of Athletic Performance, Shanghai University of Sport, Shanghai, China.; 3 Faculty of Health Sciences and Sports, Macao Polytechnic University, Macao, China.

**Keywords:** sliding sport, isokinetic strength, push technique, sport performance, biomechanics

## Abstract

The aim of this study was to explore how lower limb strength and push gait asymmetry affected performance of skeleton athletes. Isokinetic strength of the bilateral lower limb was measured in sixteen skeleton athletes. Kinematic and kinetic data were also collected during pushing. The asymmetry of lower limb strength and the push gait were measured using the symmetry angle. Asymmetry existed in the strength of the lower limbs and the push gait of skeleton athletes. The symmetry angle of peak torque of ankle dorsiflexion at 60°/s (r = −0.48, p = 0.06) and contact time (r = −0.48, p = 0.06) was moderately negatively correlated with mean push velocity, but the center of gravity height (r = 0.50, p = 0.05) and the hip joint angle (r = 0.54, p = 0.03) at the touchdown showed a moderate positive correlation with mean push velocity. The asymmetry of lower limb strength and the push gait in skeleton athletes is specialized. Reducing the difference in peak torque of dorsiflexion between both sides, as well as the disparity in contact time during the push phase, may be beneficial in enhancing push velocity.

## Introduction

Limb asymmetry refers to differences in limb structure, movement patterns or athletic abilities between the left and right sides of the body (Li et al., 2021). Limb asymmetry may arise from poor daily posture, habitual body control, long-term adaptation to specialized sports techniques, and the lack of a systematic and scientific approach to physical training, with the absence of scientific strength training and specialized sports skills being significant factors. It has been suggested that the analysis of limb asymmetry is crucial for identifying potential injury risk factors, assessing injury recovery, and optimizing physical and technical training (Li et al., 2021).

Previous studies on strength asymmetry have primarily used isokinetic strength tests (Lockie et al., 2012), mid-thigh pulls (Bailey et al., 2013, 2015), and jumping actions (Exell et al., 2017). A substantial body of research indicates that athletes in sports such as soccer (Lockie et al., 2012), basketball (Lockie et al., 2012), handball (Cadens et al., 2023), track and field (Bissas et al., 2022; Theodorou et al., 2023; Trivers et al., 2014; Vagenas and Hoshizaki, 1991), table tennis (Kalata et al., 2020), volleyball (Schons et al., 2019), and swimming (Moroucp et al., 2015; Santos et al., 2013) commonly exhibit strength asymmetry. When the degree of asymmetry exceeds a certain threshold, the likelihood of sports injuries increases. A study on hip muscle strength and hamstring injuries showed that a difference of about 10% between the lower limbs could be sufficient to cause hamstring injuries in elite sprinters, with injuries usually occurring on the weaker side (Sugiura et al., 2008).

Long-term specialized technical training and adaptation are significant factors causing asymmetry between limbs. Previous research on asymmetry in specialized techniques has primarily focused on sports such as sprinting, cycling, and swimming. Most studies support the existence of differences in sprint kinematic indicators between limbs (Girard et al., 2017; Korhonen et al., 2010; Mackala et al., 2010; Pappas et al., 2015), but findings regarding sprint kinetics are inconsistent. Research by Hamill et al. (1984) and Maupas (2002) has indicated that there are either no differences or only minor differences in kinetic indicators during sprinting between limbs. In contrast, other studies have shown that kinetic indicators do exhibit asymmetry, but with specific patterns and directions (Giakas et al., 1997; Pietraszewski et al., 2020; Raibert, 1986; Rumpf et al., 2014; Zifchock et al., 2006). Rannama et al. (2015) reported asymmetry in lower limb strength and cycling kinematics in road cyclists, finding that the highest asymmetry was in the upper body kinematics. Santos et al. (2013) and Morouco et al. (2015) conducted separate studies on the asymmetry of kinetic indicators in freestyle swimmers, with both studies showing the presence of kinetic asymmetry.

Previous research on the impact of asymmetry in limb strength and specialized technique on athletic performance is limited. Although studies have reported the presence of asymmetry in limb strength, explosive power, and the gait among many athletes, it remains unclear whether this asymmetry affects performance. Research by Trivers et al. (2014), Santos et al. (2013), and Rannama et al. (2015) has shown a negative correlation between limb asymmetry and athletic performance, whereas Lockie et al. (2012) indicate that greater differences in knee extension torque between limbs are associated with better sprint performance. Moreover, some studies suggest that asymmetry in muscle strength and movement patterns does not affect performance (Haugen et al., 2018; Maloney, 2019; Meyers et al., 2017), indicating that not all observed asymmetries are related to performance outcomes. A deeper understanding of limb asymmetry and its impact on performance is crucial for precision in physical training and refinement of specialized technical skills to enhance competitive results. Skeleton, a classic sliding event in the Winter Olympics, consists of two phases: pushing and sliding. During the push start, the skeleton athlete must maintain a forward-leaning trunk posture while rapidly alternating leg extension and swinging movements, with one arm swinging in coordination with the legs and the other arm pushing the sled forward. This raises questions about whether such movement patterns result in limb asymmetry, whether prolonged specialized training leads to differences in limb strength, and whether these differences affect performance. To date, no studies have confirmed these speculations.

Therefore, the aim of this study was to explore how lower limb strength and push gait asymmetry affected performance of skeleton athletes. The hypotheses were as follows: 1) skeleton athletes exhibit asymmetry in both lower limb strength and push technique; 2) the faster the push velocity of skeleton athletes, the lower the degree of asymmetry in the lower limb strength and push technique; 3) a positive correlation exists between the asymmetry in strength and in push technique's kinematic and kinetic indicators. The more pronounced the strength asymmetry, the greater the asymmetry seen in both the kinematic and kinetic aspects of the push technique.

## Methods

### Participants

Sixteen athletes from the Chinese national skeleton team volunteered to participate in this study, consisting of 9 male (age: 22.89 ± 0.99 years; body height: 180.67 ± 2.91 cm; body mass: 81.12 ± 4.82 kg) and 7 female athletes (age: 20.86 ± 1.46 year; body height: 171.86 ± 7.16 cm; body mass: 64.06 ± 6.95 kg). This study was carried out in accordance with the recommendations of the Science Research Ethics Committee at the Shanghai University of Sport (protocol code: 102772020RT081; approval date: 27 October 2020), with all the participants’ written informed consent, in accordance with the Declaration of Helsinki.

### Experimental Setup

Participants were required to visit the laboratory three times, with each visit spaced 2−7 days apart. In the first visit, they underwent isokinetic muscle tests for the hip, knee, and ankle joints at speeds of 60°/s and 180°/s. During the second visit, participants familiarized themselves with the push technique data collection process and practiced their steps. The final visit involved the actual collection of push technique data.

### Strength Data Collection

The IsoMed2000 isokinetic muscle testing and training system was used to assess the peak torque of concentric contractions for the hip, knee, and ankle joints of participants on both sides. Testing included slow speed (60°/s) for evaluating maximum strength of the lower limb joints and fast speed (180°/s) for assessing explosive strength. Before the official test, participants were required to perform three submaximal strength exercises to familiarize themselves with the concentric contraction movement pattern. Once the official testing commenced, concentric contractions for the flexor and extensor muscles of the hip, the knee, and the ankle were repeated five times. There was a 10-min interval between tests for each joint and a 3–5-min rest interval between tests on either side. During testing, research team members provided verbal encouragement to the participants.

### Pushing Biomechanical Data Collection

The study utilized the Vicon V5 infrared 3D motion capture system from the UK for kinematic data collection of push technique, with seven cameras placed on each side of a simulated push-start track at a 200-Hz sampling rate. Kinetic data were captured using a Kistler force plate (Switzerland), sized 90 x 60 cm, at a 1000-Hz sampling rate. Three force plates were embedded into the track’s pits, matched in dimensions, and covered with plastic mats of the same color as the track, with the Vicon system synchronously triggering the data collection.

Athletes followed a 40-min warm-up routine similar to their competition preparation, then wore spiked shoes with men wearing tight sports shorts and women wearing sports vests and tight shorts. To meet the requirements for creating a 3D human model with Visual 3D (C-Motion Inc., USA), 46 reflective markers were placed on each athlete's body. Positioned about 20 m from the first force plate, athletes started the push on command and were required to collect two valid data sets, ensuring no marker loss and continuous force plate contact by both feet. The study differentiated between the 'inside leg', the one on the same side as the pushing hand, and the 'outside leg', the opposite side.

The study processed kinematic and kinetic data using Visual 3D software, employing a 4^th^ order Butterworth low-pass filter with cutoff frequencies set at 10 Hz for kinematic data (Robertson et al., 2003) and 50 Hz for kinetic data (Riley et al., 2007). Investigated kinematic indicators included center of gravity velocity, step length, step frequency, contact time, flight time, center of gravity height, touchdown distance, the touchdown angle, take-off distance, the take-off angle, and angles of the lower limb's three joints. Kinetic indicators focused on ground reaction forces and impulse.

### Asymmetry Calculation

Asymmetry was calculated using the symmetry angle (θ_SYM_) (Zifchock et al., 2008) or all variables:


$${\text{θ}}_{\text{SYM}}=\frac{\left(45{}^{\circ}-\text{arctan}\left({\text{Χ}}_{\text{out}}/{\text{Χ}}_{\text{in}}\right)\right)}{90{}^{\circ}}\times 100\text{\%}$$

θ_SYM_ = symmetry angle value (ranging from −100% to 100%, with 0% indicating perfect symmetry

X_out_ = outside value for the variable being quantified

X_in_ = inside value for the variable being quantified

However, if: (45°− arctan(X_out_/X_in_)) >90°then it was substituted:


$${\text{θ}}_{\text{SYM}}=\frac{\left(45{}^{\circ}-\text{arctan}\left({\text{Χ}}_{\text{out}}/{\text{Χ}}_{\text{in}}\right)-180{}^{\circ}\right)}{90{}^{\circ}}\times 100\text{\%}$$

### Statistical Analysis

All results are presented as mean ± SD values. Paired-sample *t*-tests were conducted to compare differences in strength and push technique indicators between both sides of the body. To investigate the relationship between asymmetry in limb strength, push technique, and performance, it first calculated the symmetry angle for significant differences in strength and push technique indicators. Then, Pearson correlation analysis was used to explore the relationships between these symmetry angles and mean push speed. Magnitudes of correlation were classified as follows: 0 ≤ r < 0.2: “trivial”, 0.2≤ r <0.4: “small”, 0.4 ≤ r <0.6: “moderate”, 0.6 ≤ r <0.8: “strong”, and 0.8 ≤ r ≤ 1.0: “very strong” (Salkind, 2008). Statistical significance was set at *p* < 0.05.

## Results

### Strength and Pushing Asymmetries

Strength Asymmetry Results (Table 1): At a speed of 60°/s, the peak torque and relative peak torque for hip flexion in the inside leg were significantly greater than those of the outside leg. Knee extension peak torques were also higher in the inside leg, while ankle dorsiflexion peak torques were lower compared to the outside leg.

**Table 1:** Asymmetry of strength indicators for skeleton athletes.

	Outside Leg	Inside Leg	*p*	Symmetry Angle
Hip				
PT_Flex60°_ (N•m)	162.75 ± 28.49*	174.19 ± 33.71	0.05	2.06 ± 3.44
PT_Ext60°_ (N•m)	380.25 ± 69.90	379.13 ± 81.42	0.89	−0.32 ± 3.15
RPT_Flex60°_ (N•m/kg)	2.20 ± 0.21*	2.35 ± 0.28	0.04	2.06 ± 3.44
RPT_Ext60°_ (N•m/kg)	5.14 ± 0.59	5.10 ± 0.69	0.76	−0.32 ± 3.15
PT_Flex180°_ (N•m)	155.69 ± 36.41	164.94 ± 37.65	0.11	1.78 ± 4.09
PT_Ext180°_ (N•m)	334.75 ± 63.73	340.13 ± 57.99	0.32	0.62 ± 2.07
RPT_Flex180°_ (N•m/kg)	2.10 ± 0.35	2.22 ± 0.32	0.13	1.78 ± 4.09
RPT_Ext180°_ (N•m/kg)	4.52 ± 0.53	4.60 ± 0.44	0.31	0.62 ± 2.07
Knee				
PT_Flex60°_ (N•m)	137.25 ± 35.35	138.44 ± 30.72	0.74	0.51 ± 3.43
PT_Ext60°_ (N•m)	249.13 ± 61.24*	261.50 ± 53.93	0.03	1.84 ± 2.53
RPT_Flex60°_ (N•m/kg)	1.84 ± 0.31	1.86 ± 0.23	0.69	0.51 ± 3.43
RPT_Ext60°_ (N•m/kg)	3.34 ± 0.55*	3.52 ± 0.47	0.02	1.84 ± 2.53
PT_Flex180°_ (N•m)	120.94 ± 34.12	116.75 ± 27.45	0.35	−0.82 ± 4.03
PT_Ext180°_ (N•m)	178.25 ± 45.22	183.19 ± 41.40	0.11	1.07 ± 2.13
RPT_Flex180°_ (N•m/kg)	1.62 ± 0.32	1.56 ± 0.21	0.34	−0.82 ± 4.03
RPT_Ext180°_ (N•m/kg)	2.39 ± 0.39	2.46 ± 0.35	0.08	1.07 ± 2.13
Ankle				
PT_Plant60°_ (N•m)	115.13 ± 30.92	125.19 ± 31.64	0.07	1.68 ± 5.68
PT_Dors60°_ (N•m)	40.63 ± 10.28**	32.19 ± 7.12	0.00	−4.39 ± 8.46
RPT_Plant60°_ (N•m/kg)	1.55 ± 0.31	1.68 ± 0.27	0.08	1.68 ± 5.68
RPT_Dors60°_ (N•m/kg)	0.54 ± 0.09**	0.44 ± 0.09	0.00	−4.39 ± 8.46
PT_Plant180°_ (N•m)	116.13 ± 32.57	110.81 ± 29.53	0.18	−0.90 ± 4.43
PT_Dors180°_ (N•m)	34.50 ± 14.73	30.94 ± 8.93	0.34	−3.32 ± 11.17
RPT_Plant180°_ (N•m/kg)	1.56 ± 0.35	1.49 ± 0.31	0.18	1.68 ± 5.68
RPT_Dors180°_ (N•m/kg)	0.46 ± 0.16	0.41 ± 0.09	0.33	−4.39 ± 8.46

PT: peak torque; RPT: relative peak torque. * p < 0.05; ** p < 0.01

Push Kinematic Asymmetry (Table 2): Compared to the outside leg, the inside leg exhibited shorter step length, higher step frequency, longer contact time, shorter flight time, higher center of gravity height at the touchdown, longer toe-off distance, a smaller take-off angle, and greater hip and knee flexion angles at the touchdown and the end of the braking phase, with a smaller hip flexion angle at the take-off.

**Table 2:** Asymmetry of push kinematic indicators for skeleton athletes.

	Outside Leg	Inside Leg	*p*	Symmetry Angle
Step Velocity (m/s)	7.16 ± 0.65	7.11 ± 0.63	0.19	−0.21 ± 0.57
Step Length (m)	1.86 ± 0.13**	1.69 ± 0.12	0.00	−2.93 ± 1.62
Step Frequency (Hz)	4.00 ± 0.23**	4.26 ± 0.33	0.00	1.98 ± 2.25
Contact Time (s)	0.14 ± 0.02*	0.14 ± 0.02	0.03	0.86 ± 1.39
Flight Time (s)	0.12 ± 0.01**	0.10 ± 0.01	0.00	−5.53 ± 4.25
Touchdown COG Height (m)	0.75 ± 0.02*	0.76 ± 0.02	0.05	0.25 ± 0.46
Touchdown Distance (m)	−0.12 ± 0.06	−0.12 ± 0.09	0.57	−0.46 ± 28.28
Touchdown Angle (°)	80.77 ± 4.22	81.39 ± 6.13	0.51	0.20 ± 1.43
Toe-off COG Height (m)	0.77 ± 0.02	0.78 ± 0.02	0.14	0.18 ± 0.43
Toe-off Distance (m)	0.80 ± 0.06**	0.83 ± 0.08	0.01	0.96 ± 1.36
Toe-off Angle (°)	44.09 ± 1.68*	43.35 ± 2.45	0.04	−0.57 ± 0.92
Hip Angle				
When touchdown (°)	80.68 ± 9.42**	85.51 ± 9.90	0.00	1.85 ± 1.43
When braking phase finish (°)	66.56 ± 12.15**	72.58 ± 9.67	0.00	3.00 ± 3.50
When toe-off (°)	16.91 ± 11.04**	10.77 ± 8.17	0.00	−3.12 ± 23.26
Knee Angle				
When touchdown (°)	−45.00 ± 4.92	−48.97 ± 7.40	0.06	2.46 ± 4.98
When braking phase finish (°)	−49.98 ± 3.96	−54.00 ± 6.84	0.06	2.29 ± 4.40
When toe-off (°)	−10.12 ± 6.10	−10.99 ± 7.61	0.57	−1.04 ± 26.50
Ankle Angle				
When touchdown (°)	5.68 ± 5.98	7.35 ± 8.04	0.30	14.55 ± 25.84
When braking phase finish (°)	18.02 ± 4.40	18.78 ± 5.94	0.65	0.29 ± 12.20
When toe-off (°)	−35.77 ± 7.74	−35.38 ± 10.35	0.82	−1.08 ± 6.08

COG: center of gravity. * p < 0.05; ** p < 0.01

Push Kinetic Asymmetry (Table 3): The inside leg showed lower peak propulsive forces and vertical impulses compared to the outside leg.

**Table 3:** Asymmetry of push kinetic indicators for skeleton athletes.

	Outside Leg	Inside Leg	*p*	Symmetry Angle
Peak Braking Force (N/kg)	−1.82 ± 0.52	−1.86 ± 0.34	0.79	1.13 ± 7.82
Peak Propulsive Force (N/kg)	1.12 ± 0.15**	0.99 ± 0.16	0.00	−4.06 ± 4.84
Peak Vertical Force (N/kg)	24.18 ± 6.13	22.95 ± 5.50	0.34	−1.56 ± 5.88
Braking Impulse (Ns/kg)	−0.01 ± 0.00	−0.01 ± 0.00	0.70	−0.36 ± 9.68
Propulsive Impulse (Ns/kg)	0.02 ± 0.00	0.02 ± 0.01	0.82	−0.05 ± 5.96
Horizontal Impulse (Ns/kg)	0.02 ± 0.00	0.02 ± 0.01	0.70	0.19 ± 12.31
Vertical Impulse (Ns/kg)	0.54 ± 0.10**	0.49 ± 0.09	0.01	−3.06 ± 3.91

* p < 0.05; ** p < 0.01

In the strength indicators, the symmetry angle of peak torque in ankle dorsiflexion at 60°/s was the highest. In the push kinematic indicators, the symmetry angle for flight time was the largest, followed by the hip joint angles at the end of the braking phase and at the toe-off, whereas the center of gravity height at the touchdown and the toe-off angle were smaller. In the push kinetic indicators, the symmetry angle for peak propulsive force was the greatest.

### Relationships between Strength, Pushing Asymmetry and Velocity

**Figure 1:**
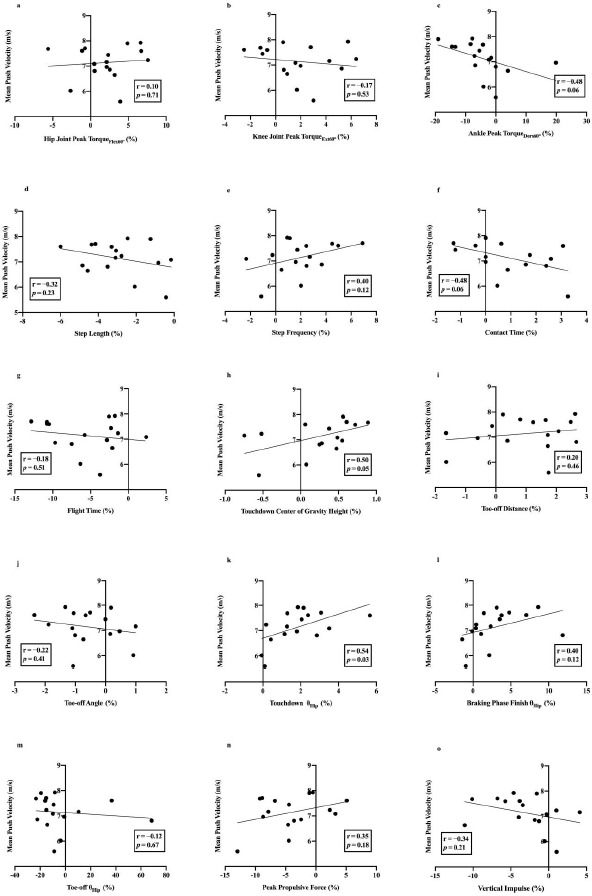
The relationships between strength, pushing asymmetry and velocity.

The relationship between lower limb strength, push technique asymmetry, and performance is illustrated in Figure 1. Analysis revealed a moderate negative correlation between the symmetry angle of peak torque of ankle dorsiflexion at 60°/s and mean push velocity (r = −0.48, *p* = 0.06). In terms of kinematic asymmetry indicators during the push phase, both the symmetry angle of the center of gravity height (r = 0.50, *p* = 0.05) and the hip joint angle (r = 0.54, *p* = 0.03) at the touchdown showed a moderate positive correlation with mean push velocity. Conversely, the symmetry angle for contact time exhibited a moderate negative correlation with mean push velocity (r = −0.48, *p* = 0.06). No significant correlation was observed between kinetic asymmetry indicators and push performance.

### Relationships between Asymmetries in Strength and Pushing

**Figure 2:**
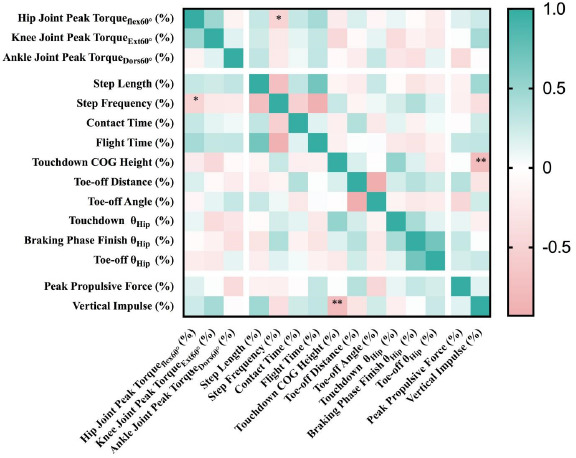
Relationships between asymmetries in strength and pushing.

The relationship between strength and push technique asymmetry is illustrated in Figure 2. A moderate negative correlation was identified between the symmetry angle of hip flexion peak torque at 60°/s and step frequency (r = −0.49, *p* = 0.05). Additionally, a strong negative correlation was observed between the symmetry angle of the center of gravity height at the touchdown and vertical impulse (r = −0.77, *p* = 0.01). No significant correlations were found between other asymmetry indicators across limbs.

## Discussion

The purpose of this study was to explore the asymmetries in lower limb strength and push technique kinematics and kinetics among skeleton athletes, as well as their relationship with performance. The results support hypothesis 1, which posits that asymmetry exists in the strength of the lower limbs and push techniques of skeleton athletes. Hypothesis 2 was partially accepted as within the strength indicators, only the symmetry angle of ankle dorsiflexion peak torque at 60°/s showed a significant negative correlation with mean push velocity (r = −0.48, *p* = 0.06), indicating that a smaller symmetry angle in ankle dorsiflexion torque correlates with faster push speeds. However, the relationship between the symmetry angle of push kinematic indicators and mean push velocity was inconsistent. The symmetry angle for contact time had a negative correlation with mean push velocity (r = −0.48, *p* = 0.06), suggesting that a smaller symmetry angle for contact time results in faster push speeds. Yet, larger symmetry angles for the center of gravity height at the touchdown (r = 0.50, *p* = 0.05) and the hip joint angle at the touchdown (r = 0.54, *p* = 0.03) correlated with faster push speeds. No significant correlation was found between the symmetry angle of kinetic indicators and mean push speed. A negative correlation was observed between the symmetry angle of hip flexion peak torque at 60°/s and step frequency, and between the center of gravity height and the vertical impulse symmetry angle, leading to the rejection of hypothesis 3.

Asymmetry exists among skeleton athletes both in terms of strength and in the kinematic and kinetic indicators of push technique. Within the strength indicators, there are evident differences between sides in the peak torque and relative peak torque for hip flexion, knee extension, and ankle dorsiflexion at 60°/s. Slow isokinetic strength tests, such as those conducted at 60°/s, are typically utilized to assess an athlete's maximum strength. Consequently, in this study, skeleton athletes exhibited significantly greater hip flexion and knee extension maximum strength and relative maximum strength in their inside leg compared to their outside leg. Nevertheless, no significant differences in hip extension strength were found between the bilateral lower limbs. This observation can be attributed to the primary centripetal contraction of the hip extensor muscle group during the early support phase, followed by a predominant centrifugal contraction to absorb energy and prepare for the end of the support phase, thereby propelling the body into the swing phase. Simultaneously, the role of the hip flexor muscle group and the knee extensor muscle group as the main sources of propulsion during the early swing phase and support phase, respectively, may contribute to this finding. Conversely, maximum and relative maximum strength in ankle dorsiflexion was significantly lower in the inside leg than in the outside leg. Limb strength asymmetry is widely observed across athletes in various sports, including team sports such as football, rugby, basketball, hockey, track and field, table tennis, volleyball, and others (Bissas et al., 2022; Schons et al., 2019; Trivers et al., 2014; Vagenas and Hoshizaki, 1991). However, some studies have indicated no significant difference in bilateral muscular strength (Maupas et al., 2002; Maloney, 2019). Research by Schiltz et al. (2009) demonstrated that the bilateral peak torque ratio in professional basketball players was higher than in young basketball players and control groups. Moreover, the asymmetry level of knee extension peak torque at 60°/s (11.3%) exceeded 10%, yet this difference was not statistically significant. They posited that years of intensive specialized training did not lead to an imbalance in the bilateral knee extension or flexion muscle groups. Contrary to this conclusion, our study suggests otherwise. During the push phase, skeleton athletes performed rapid alternating leg extensions and swings, with only one arm swinging while the other propelled the sled forward. This resulted in different range of motion for each leg. Compared to the outside leg, the inside leg demonstrated a greater angle of hip and knee flexion upon the touchdown, consequently raising the center of gravity. Conversely, the inside leg exhibited a smaller angle of hip flexion at the toe-off, leading to a smaller toe-off angle. Throughout the entire support phase, the inside leg demonstrated a wider range of hip extension and knee extension. This, combined with a shorter flight time, contributed to a shorter step length and a higher step frequency for the inside leg. Prolonged specialized push technique training without focusing on bilateral exercise variation could lead to significant differences in maximum muscle strength of hip flexion, knee extension, and especially ankle dorsiflexion between the two legs. Coaches and physical trainers should continuously monitor the strength of athletes' legs on both sides, attempting to maintain these differences within a certain range to prevent injuries.

A key finding of this study is the discovery of a notable negative correlation between the symmetry angle of peak ankle dorsiflexion torque at 60°/s and the mean push velocity. This suggests that reducing the difference in peak dorsiflexion torque between the ankles may be beneficial in enhancing push velocity. Although plantar flexion torque plays a dominant role in the ankle activity during the contact phase of the push, athletes generate a smaller dorsiflexion torque just before the toe-off, which may serve to prevent further plantar flexion of the ankle joint as it approaches the toe-off, thus reducing contact time in preparation for the flight phase. Previous research on the relationship between strength asymmetry and performance has been extensive. The findings of Trivers et al. (2014) indicate that smaller asymmetry in knee and ankle joint muscle strength is more conducive to performance. Conversely, research by Lockie et al. (2012) suggests that greater discrepancies in knee extension torque between sides correlate with faster sprint speeds, and this is compensated for by the stronger leg to overcome the difference in strength. Schons and colleagues (2019) found that differences in knee extensor muscles between sides were not related to jumping performance. In summary, most studies support the presence of strength differences in the lower limbs, but the impact of such asymmetry on performance remains inconclusive (Bishop et al., 2018).

We discovered significant asymmetries between the legs of skeleton athletes during the maximum speed push phase, including differences in step length, step frequency, contact time, flight time, the center of gravity height at the touchdown, toe-off distance, the toe-off angle, the hip joint angle at the touchdown, at the end of the braking phase and at the toe-off. There was also a trend towards significant differences in the knee joint angle at the touchdown and at the end of the braking phase. No other studies have been found that investigated asymmetries in the push technique specifically. However, most studies on sprinting, a motion similar to the push technique, support the existence of limb differences in sprinting kinematic indicators (Girard et al., 2017; Korhonen et al., 2010; Mackala et al., 2010; Pappas et al., 2015). Our study found the greatest asymmetry in the flight time (−5.53 ± 4.25%), which is consistent with previous findings that flight time shows the largest difference among lower limb kinematic indicators (Girard et al., 2017; Korhonen et al., 2010). Except for the hip joint angles at the end of the braking phase and at the toe-off, the symmetry angles for all other kinematic indicators were less than 3%. We also found no difference in step velocity between the sides, which may reduce the inefficiency of significant acceleration and deceleration between consecutive steps (Exell et al., 2017). The symmetry angle for the ankle joint angle at the touchdown was larger, but the difference between sides was not statistically significant, possibly due to high variability in ankle joint angles at the touchdown among athletes.

When addressing the relationship between sprinting asymmetry and athletic performance, most studies indicate that sprinting asymmetry is common and does not affect sprint performance (Haugen et al., 2018; Meyers et al., 2017; Maloney, 2019). Haugen and his colleagues (2018) studied the complete gait cycle of 22 elite sprinters and found that kinematic indicators of asymmetry had no relationship with performance at maximum speed phases, suggesting that bilateral asymmetry is typical in human running patterns. Contrary to these findings, our study does not support this conclusion. We discovered a negative correlation between the symmetry angle of contact time and mean push velocity in skeleton. Undoubtedly, contact time is a crucial determinant of performance (Gleadhill et al., 2021). Hence, this suggests that coaches and athletes should aim to minimize the asymmetry in contact time between sides to enhance push velocity. Furthermore, we found that the symmetry angles of the center of gravity height and the hip joint angle at the touchdown were positively correlated with mean push velocity, indicating that significant differences in the center of gravity height and the hip joint angle at the touchdown could still maintain a faster push velocity. This may imply that a smaller degree of asymmetry in the center of gravity height (symmetry angle: 0.25 ± 0.46%) and the hip joint angle (symmetry angle: 1.85 ± 1.43%) at the touchdown does not adversely affect the push velocity of faster skeleton athletes.

In this study, a significant difference between the peak propulsive force and vertical impulse in the inside and outside legs was found, yet this discrepancy did not impact the performance of the push. Previous studies on sprint kinetics have shown inconsistent results regarding the presence of differences. Research by Hamill et al. (1984) indicated that there were no limb asymmetries in kinetic determinants. This could be related to the small sample size and the fact that data collection for the left and right legs was not continuous but rather separate. Maupas et al. (2002) also demonstrated a low level of asymmetry in vertical and horizontal forces. Conversely, others have shown asymmetry in kinetic determinants, albeit with specific indicators and directions (Giakas et al., 1997; Rumpf et al., 2014; Zifchock et al., 2006). Research by Rumpf et al. (2014) highlighted that asymmetry in vertical forces was significantly greater than in horizontal forces. However, Zifchock et al. (2006) showed the least asymmetry in vertical force peaks, with a symmetry index of kinematic indicators for the lower limbs of healthy female runners at a speed of 3.7 m/s ranging from 3.1% to 49.8%. The greatest differences were observed in the peak medial and lateral ground reaction forces and peak shock. Research by Girard et al. (2017) and Korhonen et al. (2010) indicated that the degree of asymmetry in horizontal forces was much greater than in resultant or vertical forces. This study found that the degree of asymmetry in peak propulsive force and vertical impulse was greater than in other kinetic determinants. These inconsistent findings may be related to the indicators used to evaluate asymmetry, the unique background of the subjects (age, gender, training history, injury history), and the experimental design. Few studies have investigated the relationship between differences in kinetic determinants and sports performance, using asymmetry in kinetic determinants alone to explain asymmetry in kinematic indicators (Exell et al., 2017).

Apart from a moderate negative correlation between the symmetry angle of peak torque of hip flexion at 60°/s and step frequency, and a high negative correlation between the center of gravity height at the touchdown and vertical impulse, no significant correlations were found between other strength and push kinematic and kinetic asymmetry indicators. This study supports previous research on the relationship between strength and gait indicators, indicating a weak or a nonexistent relationship between the asymmetry of strength and technique kinematics and kinetics (Exell et al., 2017). Exell et al. (2017) believe that there is no relationship between kinematic asymmetry and kinetic asymmetry during the maximum speed phase in male sprinters, due to individual interactions between kinetic and kinematic asymmetries demonstrated by athletes. For some athletes, kinetic asymmetry might be a cause of certain kinematic variable asymmetries, while for others, kinetic asymmetry could reduce kinematic characteristics and might be a compensatory mechanism needed due to strength or physical imbalances.

This study is the first to examine the asymmetry of strength and push technique in skeleton athletes and their link to performance. However, it has several limitations that should be acknowledged. First, we collected push technique data on a simulated track on flat land. This setting might not fully replicate real track conditions, possibly impacting the findings. Second, the study's sample size was rather small. It included nearly all national team skeleton athletes from China, but did not categorize them by their performance level or gender. Future research should look into the asymmetry of strength and push technique on actual tracks. It should also include a larger sample size and explore differences across various performance levels and genders of athletes.

## Conclusions

The asymmetry of lower limb strength and the push gait in skeleton athletes is specialized. Reducing the difference in peak torque of dorsiflexion between both sides, as well as the disparity in contact time during the push phase, may be beneficial in enhancing push velocity. The relationship between strength and asymmetry in kinematics and kinetics is either weak or non-existent. It is recommended that coaches and strength and conditioning professionals continuously monitor asymmetry in strength and push technique of the bilateral lower limbs of skeleton athletes to minimize the risk of injury and impact on performance.
